# Assessing the sustainability of the Systems Analysis and Improvement Approach to increase HIV testing in family planning clinics in Mombasa, Kenya: results of a cluster randomized trial

**DOI:** 10.1186/s13012-022-01242-3

**Published:** 2022-10-04

**Authors:** Jessica E. Long, McKenna C. Eastment, George Wanje, Barbra A. Richardson, Emily Mwaringa, Mwanakarama Athman Mohamed, Kenneth Sherr, Ruanne V. Barnabas, Kishorchandra Mandaliya, Walter Jaoko, R. Scott McClelland

**Affiliations:** 1 Department of Epidemiology, University of Washington, 325 9th Avenue, Box 359909, Seattle, WA 98104 USA; 2 Present Address: Department of Medicine, University of Washington, Seattle, WA USA; 3 Department of Global Health, University of Washington, Seattle, WA USA; 4 Department of Biostatistics, University of Washington, Seattle, WA USA; 5 Fred Hutchinson Cancer Research Center, Vaccine and Infectious Disease Division, Seattle, WA USA; 6 Mombasa County Department of Health, Mombasa, Kenya; 7 Industrial & Systems Engineering, University of Washington, Seattle, WA USA; 8 Medical Microbiology, University of Nairobi, Nairobi, Kenya

**Keywords:** HIV counseling and testing, Family planning clinics, Implementation science, System analysis and improvement approach (SAIA), Sustainability

## Abstract

**Background:**

In Kenya, HIV incidence is highest among reproductive-age women. A key HIV mitigation strategy is the integration of HIV testing and counseling (HTC) into family planning services, but successful integration remains problematic. We conducted a cluster-randomized trial using the Systems Analysis and Improvement Approach (SAIA) to identify and address bottlenecks in HTC integration in family planning clinics in Mombasa County, Kenya. This trial (1) assessed the efficacy of this approach and (2) examined if SAIA could be sustainably incorporated into the Department of Health Services (DOHS) programmatic activities. In Stage 1, SAIA was effective at increasing HTC uptake. Here, we present Stage 2, which assessed if SAIA delivery would be sustained when implemented by the Mombasa County DOHS and if high HTC performance would continue to be observed.

**Methods:**

Twenty-four family planning clinics in Mombasa County were randomized to either the SAIA implementation strategy or standard care. In Stage 1, the study staff conducted all study activities. In Stage 2, we transitioned SAIA implementation to DOHS staff and compared HTC in the intervention versus control clinics 1-year post-transition. Study staff provided training and minimal support to DOHS implementers and collected quarterly HTC outcome data. Interviews were conducted with family planning clinic staff to assess barriers and facilitators to sustaining HTC delivery.

**Results:**

Only 39% (56/144) of planned SAIA visits were completed, largely due to the COVID-19 pandemic and a prolonged healthcare worker strike. In the final study quarter, 81.6% (160/196) of new clients at intervention facilities received HIV counseling, compared to 22.4% (55/245) in control facilities (prevalence rate ratio [PRR]=3.64, 95% confidence interval [CI]=2.68–4.94). HIV testing was conducted with 60.5% (118/195) of new family planning clients in intervention clinics, compared to 18.8% (45/240) in control clinics (PRR=3.23, 95% CI=2.29–4.55). Interviews with family planning clinic staff suggested institutionalization contributed to sustained HTC delivery, facilitated by low implementation strategy complexity and continued oversight.

**Conclusions:**

Intervention clinics demonstrated sustained improvement in HTC after SAIA was transitioned to DOHS leadership despite wide-scale healthcare disruptions and incomplete delivery of the implementation strategy. These findings suggest that system interventions may be sustained when integrated into DOHS programmatic activities.

**Trial registration:**

ClinicalTrials.gov (NCT02994355) registered on 16 December 2016.

Contributions to the literature

- Sustainability is an under-measured aspect of implementation studies and is critical to understanding the lasting impact of effective interventions.
- We measured the sustainment of both an evidence-based intervention and an implementation strategy, and barriers and facilitators to sustainment.
- In this setting, where a county-level Department of Health Services (DOHS) oversees health facilities, the evidence-based intervention was sustained after embedding the implementation strategy into DOHS’s routine programmatic activity.
- Institutionalization, or establishment of the evidence-based intervention as normal practice, was facilitated by the low complexity of the implementation strategy, positive implementation climate within facilities, and a health system structure that allowed DOHS-led oversight and cost coverage.

## Introduction

Eastern and southern Africa have seen remarkable reductions in HIV incidence, but gender gaps still remain [1]. In 2019, two in five new infections in this region were among women, and adolescent girls and young women (aged 15 to 24 years) were 2.5 times more likely than male peers to acquire HIV [1]. In Kenya, 6.6% of women are living with HIV, and the incidence is highest among young women of reproductive age [2]. Effective outreach, testing, and linkage to care among women are essential to reduce the burden and spread of HIV and achieve the United Nations Joint Program on HIV/AIDS (UNAIDS) 95-95-95 goal of 95% of people living with HIV knowing their status, 95% antiretroviral therapy (ART) use among those diagnosed with HIV, and 95% viral load suppression among those on ART. A key strategy for reaching women of reproductive age and improving HIV testing uptake is the integration of HIV testing and counseling (HTC) into family planning services [3–5]. However, while many countries have national guidelines to support HTC at family planning clinics, implementation of this intervention varies widely between and within countries. In Kenya, HTC integration into family planning services is promoted by Kenya’s National AIDS and STD Control Program, but successful integration of these services remains low in many regions, including Mombasa County [5, 6].

Implementation strategies, such as the Systems Analysis and Improvement Approach (SAIA), can be used to systematically identify and address bottlenecks in healthcare delivery systems. SAIA is an evidenced-based multi-component implementation strategy that is applied at the facility level to provide staff with tools aimed at improving care cascades. Through a five-step process (described below) SAIA provides a system-wide view of a healthcare cascade and uses small tests of change to address context-specific bottlenecks and barriers to care delivery [7]. Using this method, SAIA provides healthcare teams with tools to collaboratively identify problems, prioritize areas of improvement, implement changes, and evaluate those changes [8, 9]. This method is theorized to improve service-delivery outcomes by facilitating communication, promoting consensus decision-making, and encouraging accountability across staff within a care cascade [8, 10]. SAIA has previously been tested as a strategy to improve healthcare cascades focused on the prevention of mother-to-child HIV transmission [11], mental healthcare [8], and integrating hypertension diagnosis and management into the HIV care cascade [10], among others. An important topic of the ongoing study is the sustainability of these interventions and the impact they have on long-term changes in delivery systems after the research has ended.

While there is no standard definition of sustainment in implementation research [12, 13], it is often conceptualized as the continued use of program components and activities to achieve desirable program and population outcomes over time [12, 14, 15]. This can encompass both continued adherence to the implementation strategy (e.g., SAIA) and continued delivery of the evidence-based intervention (e.g., HTC) [12]. For interventions delivered within healthcare settings, an important aspect of sustainability is how well the implementation strategy or evidence-based intervention was integrated into normal activity.

To assess the effectiveness and sustainability of SAIA as a strategy to increase uptake of HTC at family planning clinics, we conducted a two-stage cluster-randomized trial in Mombasa, Kenya. In the first stage of the trial, the study staff implemented SAIA at family planning clinics in the intervention arm for 1 year. At the end of the first stage, 85% (740/868) of new family planning clients were counseled about the need to complete opt-out HIV testing in intervention clinics compared to 67% (1036/1542) in control clinics (prevalence rate ratio [PRR]: 1.27, 95% confidence interval [CI], 1.15–1.30) [16]. Testing was conducted among 42% (364/859) of new clients at intervention clinics compared to 32% (485/1521) at control clinics (PRR: 1.33, 95% CI, 1.16–1.52). These results showed that SAIA was effective in increasing rates of both pre-test counseling and HIV testing at clinics in the intervention arm compared to control clinics.

Here, we present the results of the second stage of the trial. At this stage, we were interested in determining the feasibility of transitioning SAIA to Mombasa County leadership to integrate SAIA implementation into standard county oversight of family planning clinics. We hypothesized that providing continued oversight to family planning clinics through an existing county oversight mechanism would provide structure and motivation to continue SAIA implementation and that this could contribute to the long-term sustainment of the higher HTC levels observed in the first stage of the trial. To test this, we transitioned SAIA implementation and leadership to the Mombasa County Department of Health Services (DOHS), tracked the frequency of county-led SAIA visits, and compared HTC in clinics in the intervention arm versus the control arm of the trial 1 year after this handover. The aim of this stage of the trial was to assess if both SAIA delivery, and the observed improvements in uptake of HTC in family planning clinics would be sustained when implemented by the county with minimal support from study staff.

## Methods

### Study design and randomization

This study was a two-stage cluster-randomized trial to evaluate the use of SAIA to improve HTC at family planning clinics in Mombasa, Kenya. The trial design and Stage 1 results have been previously reported [16]. In brief, SAIA is a blended implementation strategy that iteratively uses a 5-step cycle to improve performance across care cascades [11]. Twenty-four family planning clinics in Mombasa were selected for study inclusion and randomized to either receive the SAIA implementation strategy (*n*=12) or to be included as controls receiving standard care (*n*=12). Restricted randomization of clinics (1:1) was conducted based on clinic size and delivery of HTC services prior to the study start. Due to the nature of the implementation strategy delivered to clinics in the intervention arm, participating clinics were not blinded. Randomization was conducted by an independent statistician at the Center for AIDS Research Biometrics Core at the University of Washington who did not serve in any other role in the study.

### County DOHS collaborators

Kenya has a decentralized system of government, in which the national Ministry of Health (MOH) provides policy, but each county independently operates a County Department of Health Services. In Mombasa County, an Executive of Health oversees two branches, Public Health and Medical Services, which are each led by a Chief Officer and Director. Under this leadership, the County Health Management Team (CHMT) operates across both branches, divided into departments to address important health topics. For this research, we worked with the Reproductive Health (RH) Officer and HIV/Sexually Transmitted Infections (STI) Officer. These officers oversee sub-county RH and STI coordinators, who have direct oversight over family planning clinics within their respective sub-counties. The sub-county STI and RH coordinators are primarily nurses and clinical officers by training who have risen to a supervisory role through years of service and professional development. Their primary role is to supervise the delivery of STI and RH services, respectively, in their sub-county jurisdictions. As part of their standard duties, RH and STI coordinators visit family planning clinics monthly to address any problems and track the progress of programmatic activities. All study activities were conducted in coordination with collaborators within the Mombasa County DOHS.

### Stage 1: Study setting

Trial Stage 1 was conducted from December 2018 to November 2019. During this time, Mombasa County did not experience any systematic disruptions to the healthcare system. At the study start, Mombasa had approximately 170 family planning clinics, including public and private facilities. All facilities receive HIV-testing supplies at no cost from the Mombasa County DOHS. HIV-testing commodities are tracked on MOH-provided registers. Specific training and certification are required to perform HTC. In the context of this study, the counseling aspect of HTC refers to pre-test counseling, in which care providers recommend opt-out HIV testing and ask family planning clients if they are willing to be tested. Anyone who reports a previous HIV-positive diagnosis is not eligible for HIV testing. For each family planning client, MOH-provided registers record if they received this counseling, HIV serostatus at the time of counseling, and if they received HIV testing. These data were used to calculate HTC rates in clinics in the intervention arm versus control clinics, with all new family planning clients considered eligible for counseling, and all new clients who did not have a previous HIV-positive diagnosis eligible for testing.

### Stage 1: Procedures

During Stage 1 of the trial, study staff implemented SAIA at each clinic in the intervention arm. The SAIA steps and roles played by the clinic staff and facilitators are explained in Table 1. As previously described [16], this included the creation of a “cascade analysis tool,” an Excel-based system for quantifying and displaying the number of individuals who complete each step of a process to identify where improvement may be needed [9, 17]. The tool also shows the expected impact on HIV testing when each step of the cascade is optimized to full performance. Cascade analysis was followed by sequential process flow mapping, in which study staff helped clinic staff to map clinic processes to identify modifiable bottlenecks in their workflow for HTC. Study staff then worked with clinic staff to conduct plan-do-study-act (PDSA) cycles, in which they identified workflow modifications that clinic staff would implement during the following month (termed “micro-interventions”) chosen to address barriers to implementing HTC specific to each clinic, then evaluate during the following cycle. Study staff conducted monthly SAIA visits with clinic staff at facilities in the intervention arm to assess the implementation and impact of the micro-interventions with real-time data input into the cascade analysis tool and plan a micro-intervention for the next month. Micro-intervention activities were enacted by the clinic staff at each family planning clinic over the following month. Examples of micro-interventions implemented in Stage 1 have been previously published [16]. Research staff conducted monthly SAIA visits for 12 months at each participating clinic in the intervention arm, during which time study data on HTC outcomes were also collected.

**Table 1:** Description of the 5-step SAIA cycle conducted with intervention clinics

SAIA step	Description	Frequency and location	FP clinic role	Facilitation
Cascade analysis tool	Excel-based system for quantifying and displaying the number of individuals who complete each step of a process to identify where improvement may be needed	Monthly SAIA meetings at clinics in the intervention arm	Clinic managers review the results of the cascade analysis monthly with study staff facilitators (Stage 1) or DOHS facilitators (Stage 2)	Facilitators populate the cascade analysis tool, and review results with FP clinic staff. Facilitators were study staff in Stage 1, DOHS staff in Stage 2.
Sequential process flow mapping	Drawn map of clinic processes and client movement through clinics to identify modifiable bottlenecks in their workflow for HTC	At initial training and at monthly SAIA meetings at clinics in the intervention arm	FP clinic staff draw the map of their specific clinic flow	Facilitators trained FP clinic staff and oversaw process mapping.
Micro-intervention setting	Identification of workflow modifications (termed “micro-interventions”) that clinic staff implement during the following month chosen to address clinic-specific barriers to implementing HTC	Monthly SAIA meetings at clinics in the intervention arm	FP clinic managers develop a micro-intervention that addresses a problem specific to their clinic, then implement that change over the following month.	Facilitators assist in developing ideas for feasible micro-interventions.
Micro-intervention assessment	Micro-intervention chosen in the previous monthly SAIA meeting is assessed to determine if it was (a) successfully implemented, (b) effective in improving HTC, and (c) if the micro-intervention should be adapted, adopted, or abandoned.	Monthly SAIA meetings at clinics in the intervention arm	FP clinic managers report on how successfully the micro-intervention was implemented, and decide if it should be adapted, adopted, or abandoned.	Facilitators provide HTC updates based on the Cascade Analysis Tool and assist FP clinic managers in decision making on next steps.
Iterative refinement	All previous steps are repeated at monthly SAIA meetings, where previous micro-interventions are reviewed, and new ones are chosen.	Monthly SAIA meetings at clinics in the intervention arm	FP clinic managers meet with facilitators and are prepared to review previous micro-interventions and set new ones.	Facilitators arrange SAIA meetings and attend monthly.

Control clinics were aware of the study but did not receive any of the SAIA implementation strategy components described above and instead continued with standard care and delivery of HTC. Kenya MOH National Guidelines recommend integration of HTC at family planning clinics, and this is overseen by DOHS RH and STI coordinators [18]. However, no specific strategies are in place to promote HTC uptake at these clinics. Study staff visited control clinics every 3 months to collect data, but otherwise had no interaction with control clinic staff. During this stage, the County DOHS leadership were updated regularly regarding study activities and served in an advisory role.

At the end of study Stage 1, there was a brief gap in the trial before Stage 2 was launched. During this time, study staff continued to actively deliver SAIA at clinics in the intervention arm.

### Power and sample size

Sample size estimates are based on Stage 1 of the trial and have been described previously [16]. Briefly, sample size determination was made based on an average of 15 new family planning clients per clinic per 3-month period, 20% HIV testing among new clients in the control clinics, and a 50% increase in HTC with the SAIA implementation strategy. At an alpha level of 0.05 and a two-sided test, the inclusion of 11 clinics per study arm would provide 80% power to detect this effect in clinics in the intervention arm compared to control clinics. To allow for potential loss to follow up of one clinic per arm, 24 clinics were randomized. Twenty-three clinics remained in follow-up throughout both stages of the study.

### Stage 2: Study setting

Trial Stage 2 was conducted from February 2020 to January 2021. In March 2020, the first cases of COVID-19 were detected in Kenya. This led to government-mandated restrictions beginning March 18, 2020, including curfews, travel restrictions, school closures, bans on gatherings of >15 people, and drastic restrictions on public transportation. In Mombasa County, a full lockdown was issued for some areas between May 6 to July 7. In October and November 2020, healthcare workers in Mombasa began a “go-slow” period, in which healthcare services were restricted to emergencies only, followed by a full strike from December 28, 2020, to February 19, 2021. These events resulted in temporary closures of some family planning clinics, reduced staff and capacity at public clinics that remained open, and reduced capacity at the county level to oversee family planning clinics. While these events were disruptive to care delivery, the purpose of Stage 2 of the trial was to assess if both SAIA delivery and higher HTC performance in family planning clinics would be sustained when implemented by the County in real-world circumstances, so it did not impact the timeline of data collection for this study.

### Stage 2: Procedures

In study Stage 2, clinics maintained the study arm that they were randomized to in the first year of the study. To support the transition of SAIA implementation to the Mombasa County DOHS, the research team trained 16 sub-county RH and STI Coordinators who were appointed by the RH and HIV/STI Officers as “implementers” to conduct SAIA visits at each clinic in the intervention arm as part of their normally scheduled monthly family planning clinic supervision visits. Four teams of STI and RH Coordinators conducted SAIA visits at all clinics in the intervention arm within their coverage area (between 1 and 6 clinics per team, depending on coverage area).

Trainings led by study staff provided the sub-county RH and STI Coordinators with an overview of SAIA, practice in collecting and recording data, and mock SAIA visits. The implementers were then responsible for traveling to assigned clinics in the intervention arm for SAIA visits to conduct or update flow mapping (as necessary), cascade analysis, and development and assessment of micro-interventions. The targeted SAIA visit schedule was one visit per month to each clinic for 12 months. The initial Stage 2 SAIA cycle at each clinic was conducted by the County implementers with oversight and mentorship from the study staff. After this initial mentored hand-off, study staff were available to answer questions and conducted periodic check-ins to monitor progress. Aside from the initial mentored cycle, the study staff did not participate in the implementation of SAIA during Stage 2 of the trial. County implementers were provided tablets preloaded with training materials that they used for data collection at sites. No other funding support or incentives were given by the study to complete these visits. County implementers reported to study staff when each SAIA visit was completed to allow study staff to track when DOHS-led SAIA cycles were conducted at each site. To avoid behavior changes induced by observation, study staff did not attend any additional SAIA cycle meetings after the mentored cycle, and therefore, we were unable to collect data on potential adaptations made at SAIA visits led by DOHS staff.

Three types of data were collected during this stage of the study. First, DOHS-appointed implementers recorded information about each SAIA visit in a REDCap questionnaire, which provided fidelity data on completion of SAIA cycles. Second, the clinical outcomes of interest (number of new family planning clients, number counseled, number tested) were independently collected quarterly by study staff directly from register data at each family planning clinic. Third, interviews were conducted with clinic staff and managers to assess barriers and facilitators to uptake and sustainment of HTC and SAIA, and reflections on why improvements were or were not sustained at their clinic.

### Participants

Data for this study were collected at the clinic level. Family planning clinic staff and managers worked with the STI and RH coordinators to implement SAIA at each clinic and participated in exit interviews at the end of the study. This study did not involve direct contact with family planning clients, and all client data were de-identified and aggregated.

### Outcomes

Outcomes of interest included sustained delivery of SAIA and maintenance of improvements in HTC observed in Stage 1 of the trial. Sustained delivery of SAIA was measured as the proportion of the planned monthly SAIA visits that were completed. Interviews with clinic staff were used to provide context for gaps in SAIA delivery. Interviews also assessed institutionalization of HTC, as well as facilitators and barriers that impacted sustainment of SAIA implementation and HTC at clinics in the intervention arm.

HTC delivery was measured as the proportion of new family planning clients tested for HIV in the final 3 months of the study, comparing clinics in the intervention arm to control clinics. A secondary measure of continued HTC was the proportion of new family planning clients who received pre-test counseling in the final 3 months in clinics in the intervention arm compared to controls. These outcome variables were collected quarterly even if no SAIA visits had been conducted and were recorded as zero new family planning clients during temporary clinic closures.

### Statistical analysis

The primary analysis followed an intent-to-treat design based on the arm of the trial each clinic was randomized to, regardless of participation in SAIA implementation procedures. Study data were collected each quarter (Q) of the 2-year study, with Stage 2 data collected in Q5–Q8. We calculated prevalence rate ratios (PRR) using Poisson regression with a log link, comparing the rates of HIV testing in the final 3 months of Stage 2 (Q8) in clinics in the intervention arm versus control clinics. A secondary analysis used the same method to compare rates of pre-test counseling in the intervention arm compared to the control. For both outcomes, we also examined if performance differed between public and private family planning clinics. Results were stratified by public versus private family planning clinics if an interaction term *p* value was <0.05. As an exploratory analysis, we further examined HTC rates over the course of Stage 2 using a difference-in-differences analysis in which we compared the change in HIV testing and counseling rates from Q5 to Q8 at clinics in the intervention arm versus control clinics. All analyses used Stata version 15.1 (College Station, TX, USA, 2017).

All clinics in the intervention arm (*n*=12) were invited to participate in exit interviews after study Stage 2 was complete. An interview guide was developed using adapted measures from the Consolidated Framework for Implementation Research (CFIR) interview guide tool (https://cfirguide.org/) and a psychometrically validated tool developed by Weiner et al. [19]. Interviews were conducted among family planning clinic staff members who were involved in SAIA implementation, and were analyzed using a rapid assessment approach guided by the CFIR [20] and the Implementation Outcomes Framework [21]. This analysis includes interview responses focused on barriers and facilitators of sustaining the implementation strategy (SAIA) and the evidence-based intervention (HTC), with a specific focus on CFIR domains of intervention characteristics, inner setting, and process, as well as concepts specific to sustainability, such as institutionalization. Interviews were recorded through field notes and audio recordings (GW). A structured codebook was created to allow for categorization of elicited constructs and emergent themes and was populated by two coders using field notes (GW, JL). Interpretation of coding was discussed iteratively between the two coders until consensus was reached.

### Ethical considerations

This research was approved by the Kenyatta National Hospital-University of Nairobi Ethics and Research Committee, the Human Subjects Research Institutional Review Board at the University of Washington, and the Mombasa County DOHS. This trial is registered at ClinicalTrials.gov (NCT02994355). All Mombasa County DOHS implementers verbally agreed to participate in the trial, and clinic staff and managers who participated in interviews provided written informed consent prior to COVID-19 and verbal assent for remote interviews during the pandemic.

## Results

**Fig. 1:**
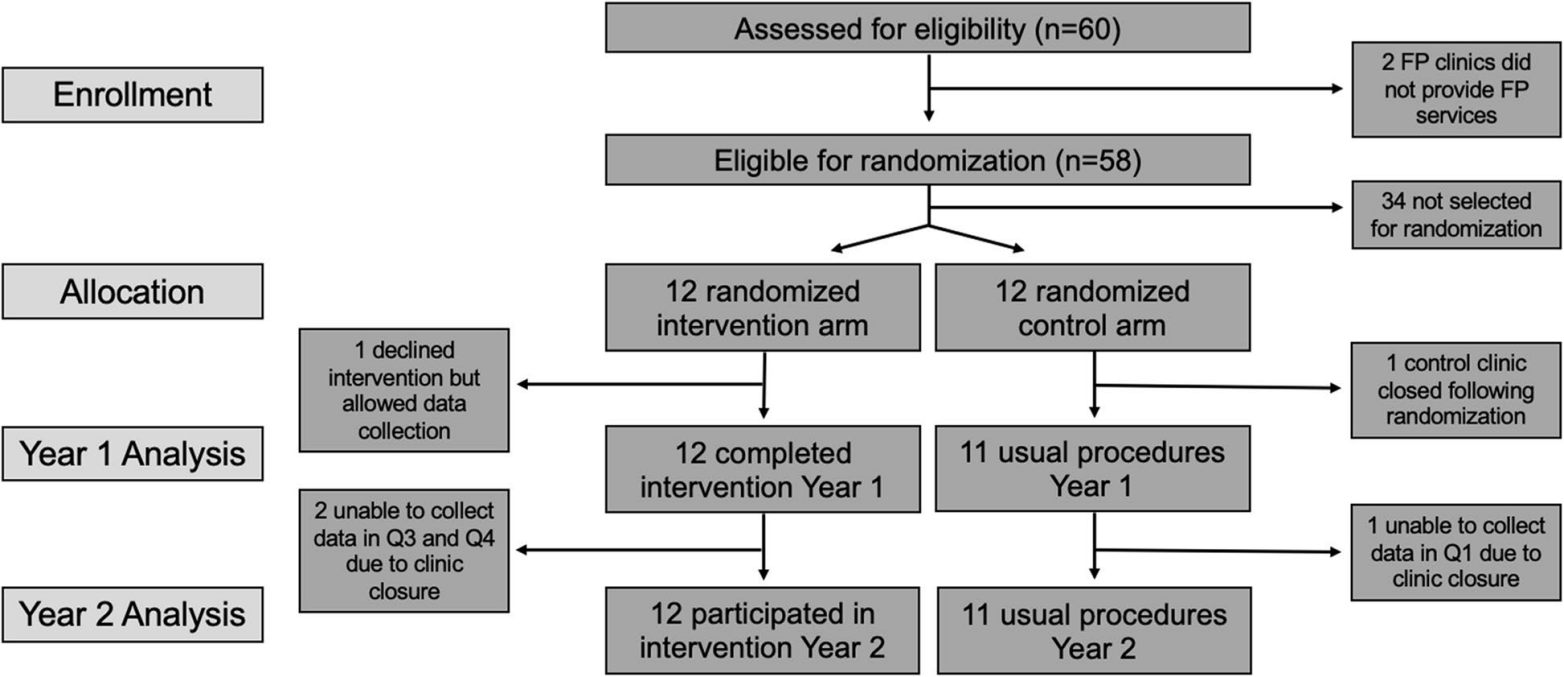
Flow diagram of family planning clinics. Flow diagram of family planning clinics assessed for eligibility, randomized, participated, and included in final intent-to-treat analysis in the first and second year of the study

Of the 24 randomized clinics, 23 contributed data to Stage 2 outcomes (Fig. 1); one control clinic closed prior to the start of the trial. In each arm of the trial, 6 clinics (50%) were public, and 4 clinics (33%) were in an urban location. In both control arm and intervention arm clinics, a median of 1 (interquartile range [IQR] 0–2) providers were trained in HTC at study baseline. In both study arms, a median of 1 (IQR 0–2) family planning clinic manager reported awareness of the most recent National HIV Guidelines at study baseline.

The COVID-19 pandemic and subsequent healthcare worker strike caused short-term clinic closures in some participating clinics. One control clinic was unable to contribute Q5 data due to temporary closure, and two clinics in the intervention arm were closed and unable to contribute data during Q7 and Q8. Additionally, one clinic in the intervention arm declined participation in SAIA implementation but allowed data collection and is included in analyses.

### HIV testing and counseling

**Fig. 2:**
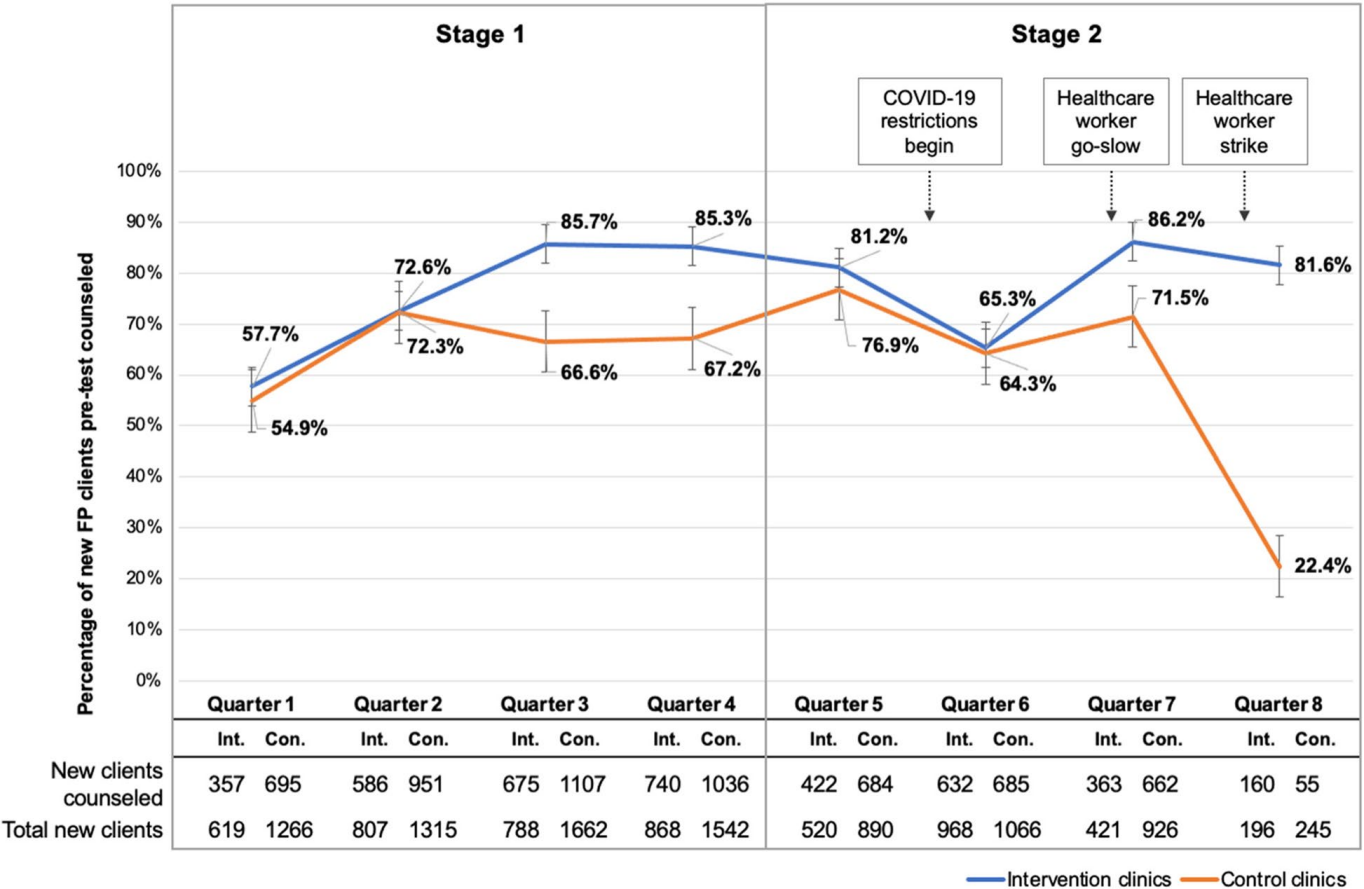
Proportion of eligible family planning clients receiving pre-test HIV counseling. Pre-test HIV counseling by quarter for the entire duration of the study. Results from Stage 1 have been previously published [16] and are re-presented here for context. Error bars reflect the standard error. (FP family planning, Int. intervention arm, Con. control arm)

**Fig. 3:**
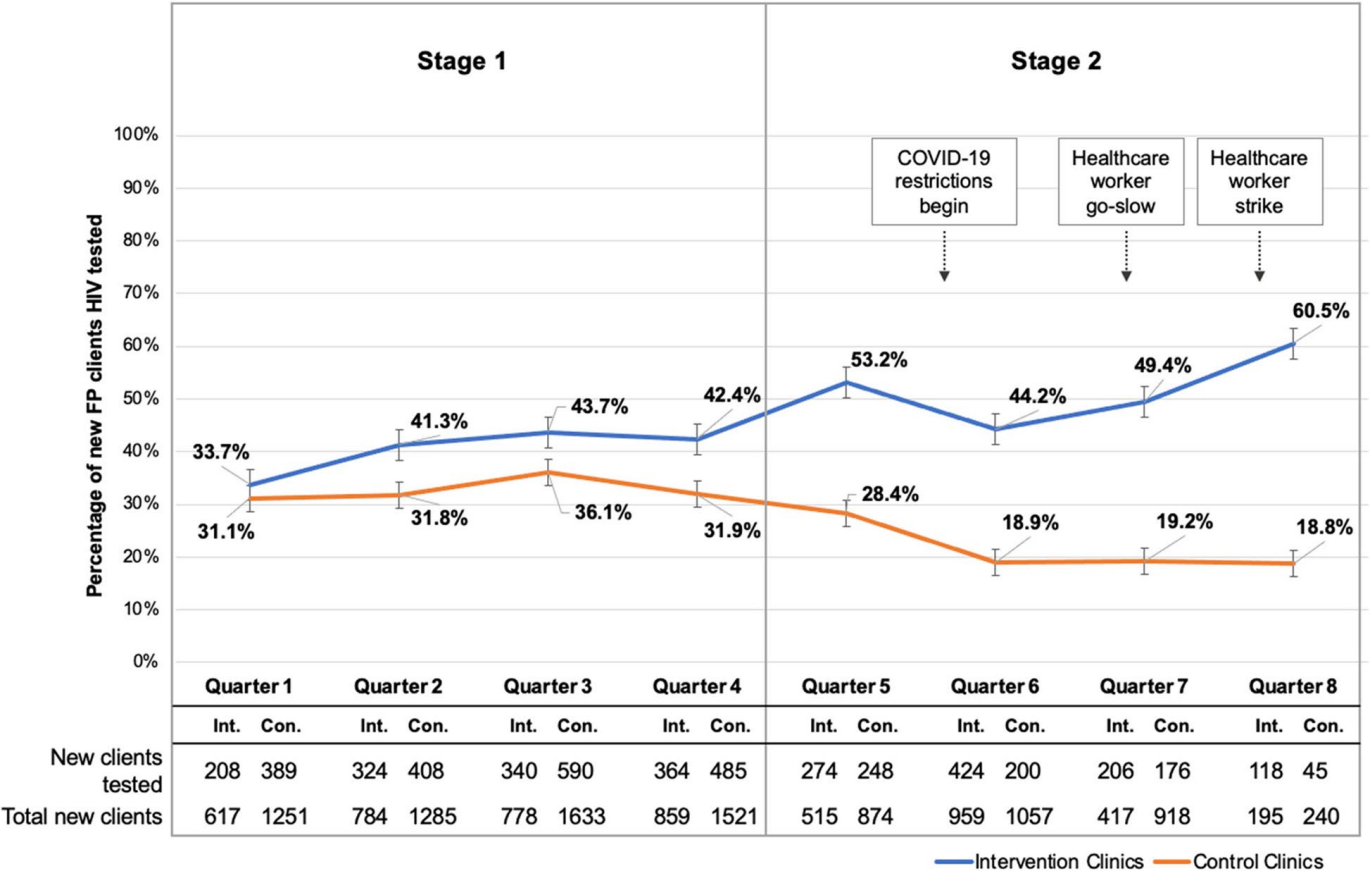
Proportion of eligible family planning clients tested for HIV. HIV testing by quarter for the entire duration of the study. Results from Stage 1 have been previously published [16] and are re-presented here for context. Error bars reflect the standard error. (FP family planning, Int. intervention arm, Con. control arm)

Stage 1 results reviewed in the introduction of this paper have been previously published [14] and are incorporated in Figs. 2 and 3 to provide context. In Stage 2, 5232 new family planning clients were seen at all clinics during the 12 months of data collection, with a precipitous drop in new clients observed in Q8 resulting from the healthcare worker strike and subsequent clinic closures (Fig. 2, bottom row). Clinics in the intervention arm (*n*=10) saw a median of 18 (IQR 11–20) new family planning clients per clinic in Q8, compared to a median of 7 (IQR 5–30) in control clinics (*n*=11).

**Fig. 4:**
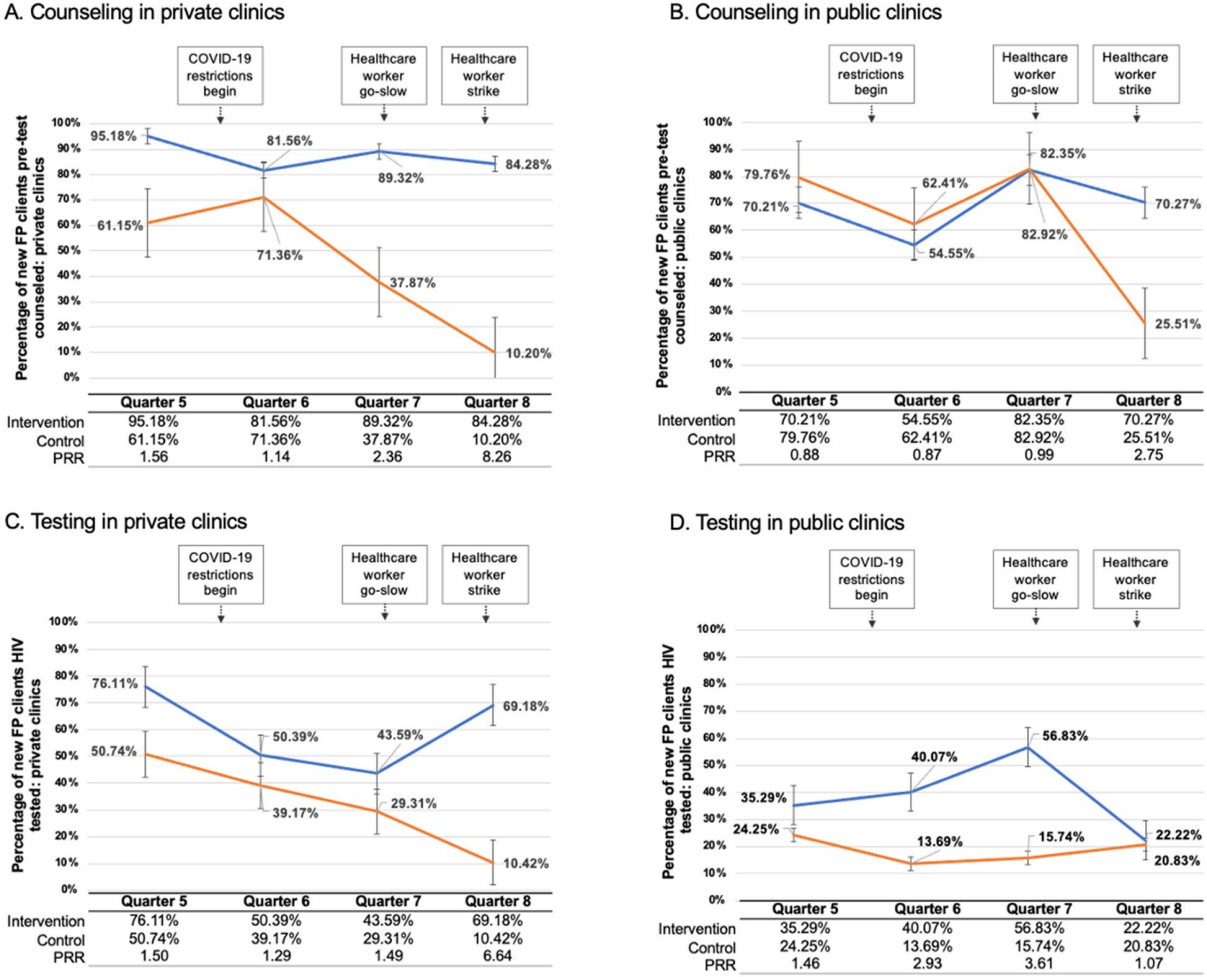
Proportion of eligible family planning clients receiving HTC in public versus private clinics. **A** Proportion of eligible family planning clients at private health facilities (*n*=11) receiving pre-test HIV counseling from Q5 to Q8 of the trial in intervention and control clinics. **B** Proportion of eligible family planning clients at public health facilities (*n*=12) receiving pre-test HIV counseling from Q5 to Q8 of the trial in intervention and control clinics. **C** Proportion of eligible family planning clients at private health facilities (*n*=11) tested for HIV from Q5 to Q8 of the trial in intervention and control clinics. **D** Proportion of eligible family planning clients at public health facilities (*n*=12) tested for HIV from Q5 to Q8 of the trial in intervention and control clinics. Error bars reflect the standard error. (FP family planning, PRR, prevalence rate ratio)

In Q8, pre-test counseling was conducted with 81.6% (160/196) of new family planning clients at intervention arm facilities compared to 22.4% (55/245) in control arm facilities (PRR 3.64, 95% CI 2.68–4.94) (Fig. 2). This effect was modified by clinic type, with a strong effect of the SAIA implementation strategy found in private clinics (PRR 8.26, 95% CI 3.38–20.17) and a smaller but still highly significant effect in public clinics (PRR 2.75, 95% CI 1.71–4.42) (Fig. 4). A difference-in-differences analysis showed a significant difference between clinics in the intervention arm compared to control clinics from Q5 to Q8. Clinics in the intervention arm saw a 0.5% increase in HIV counseling comparing Q5 to Q8, while pre-test counseling decreased in control clinics by 54% during the same time period, for a difference in differences of 54.5% between intervention arm and control arm clinics (*p*<0.05).

In Q8, 60.5% (118/195) of new family planning clients were tested for HIV in clinics in the intervention arm, compared to 18.8% (45/240) in clinics in the control arm (PRR 3.23, 95% CI 2.29–4.55) (Fig. 3). Similar to counseling, this effect was strongly modified by clinic type; private clinics in the intervention arm had a significantly higher rate of HIV testing in new family planning clients compared to private clinics in the control arm (PRR 6.64, 95% CI 2.71–16.27) (Fig. 4). In contrast, no effect of the SAIA implementation strategy was seen in the comparison of public intervention arm versus control arm clinics (PRR 1.07, 95% CI 0.50–2.28). A difference-in-differences analysis comparing the difference in HIV testing rates from Q5 to Q8 in each study arm found that HIV testing increased 7% in clinics in the intervention arm, while it decreased 10% in control clinics, corresponding to a difference in differences of 17% (*p*<0.05).

In Stage 2 of the study, there were 3/2105 (0.14%) new HIV diagnoses at intervention clinics, compared to 3/3127 (0.10%) at control facilities.

### SAIA fidelity

**Fig. 5:**
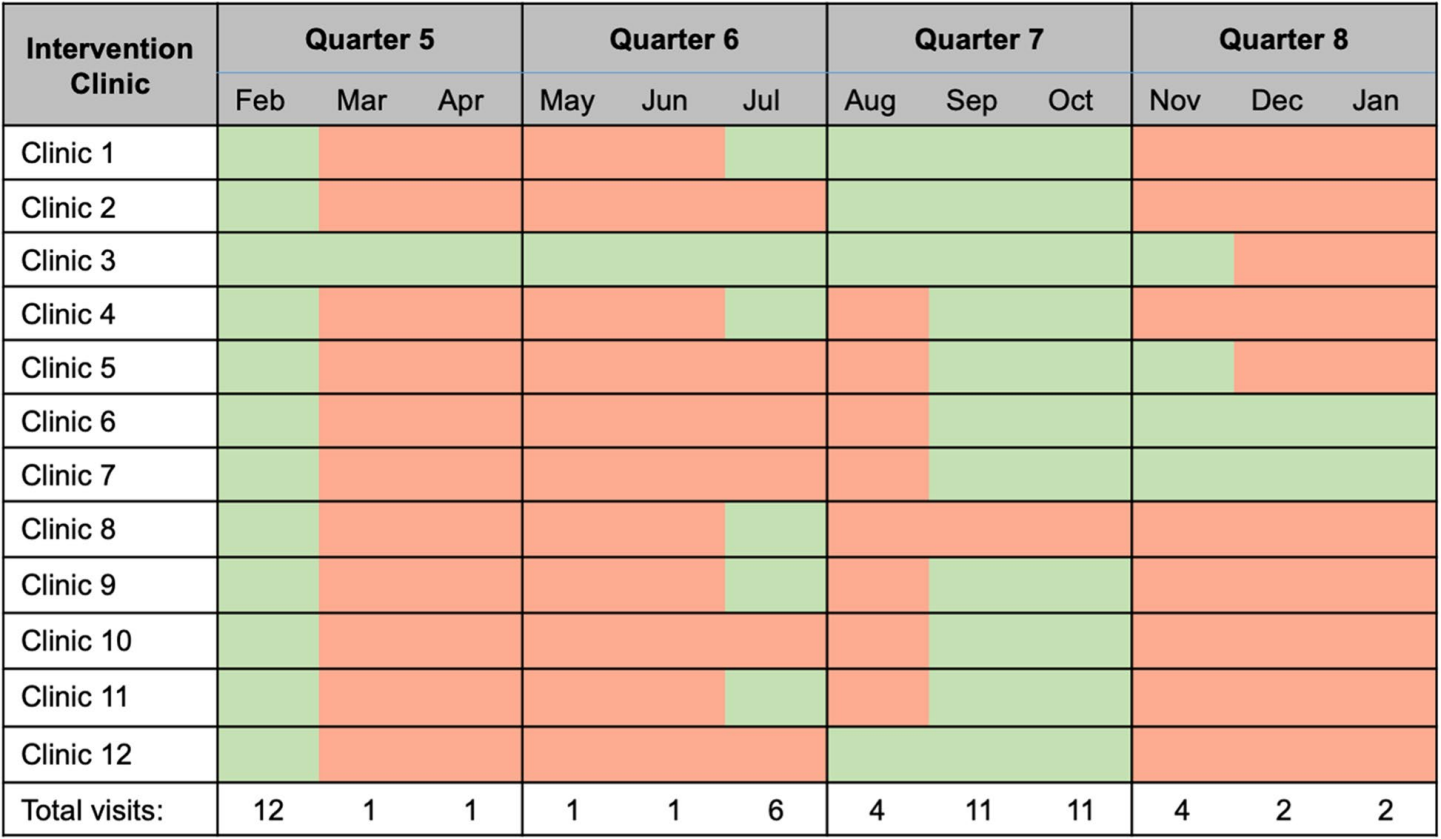
Schedule of SAIA monthly visits that were completed by the supervising sub-county implementer during Stage 2 follow-up, from February 2020 to January 2021. Green indicates when visits did occur and red indicates that a supervising sub-county STI or RH coordinator did not visit the clinic. This visit schedule reflects the context in Mombasa County at the time of the study. In February 2020, before any COVID-19 cases were reported in Kenya, the sub-county Coordinators completed supervised hand-off visits with study staff. In March 2020, the COVID-19 pandemic reached Kenya, and restrictions were put in place that impacted government-run services. Beginning in July, normal operations returned to a certain extent, and SAIA supervision visits resumed. However, the healthcare worker go-slow began in October 2020, followed by a full healthcare worker strike beginning in December 2020 that lasted through the end of the study and resulted in disruption of study activities.

Optimal delivery of the SAIA implementation strategy would require monthly visits at each clinic in the intervention arm, for a total of 144 visits performed by STI and RH Coordinators. However, due to the COVID-19 pandemic and healthcare worker strike, formal SAIA cycles with oversight from DOHS supervisors were not completed monthly at all family planning clinics in the study. Overall, 39% (56/144) of DOHS-led SAIA visits occurred, and no clinic completed all DOHS-led SAIA visits (Fig. 5). STI and RH coordinators reported at least one visit to oversee a SAIA cycle at 12 clinics in Q5, 6 in Q6, 11 in Q7, and 4 in Q8. Due to the nature of this phase of the research, study staff did not have contact with clinics, and therefore, they were unable to assess the degree to which clinics in the intervention arm conducted elements of the SAIA implementation strategy (e.g., implementing micro-interventions) in the months that the County was not available to oversee visits. Study staff were also unable to assess if County implementers made adaptations to the SAIA approach when they conducted SAIA visits.

### Barriers and facilitators to sustainment

Participants from seven clinics in the intervention arm took part in the interviews; five intervention arm clinics were unable to participate due to the timing of the interviews during the COVID-19 pandemic. During exit interviews, staff at clinics in the intervention arm provided insight into why they believed their clinics maintained high levels of HTC despite healthcare interruptions and inconsistent implementation of SAIA. Institutionalization of HTC due to the routine use of SAIA was a common theme in all interviews. Reported barriers and facilitators of SAIA implementation mapped to the CFIR constructions of complexity, implementation climate, and leadership engagement. In addition, emergent themes were well aligned with recently proposed new constructs to adapt CFIR to research in low- and middle-income countries [22]. These themes included systems architecture and perceived sustainability.

Several responses from interviewed staff suggested institutionalization of HTC, which they attribute to the impact of SAIA. These responses suggested that HTC was conducted by all staff within facilities as a part of their regular work stream, and SAIA was viewed as an ongoing activity to promote continued HTC:

It is everyday work. Now we are doing what is expected of us. The guidelines say we counsel and test. Most people [at other facilities] are not doing that. SAIA helped reach testing targets... We will continue with SAIA because it helps reach our targets for the facility. Nurse in-charge, Rural public clinic

We encourage nowadays all our FP clients to know their status. Every staff is doing that from [clinical officer] CO to me in the lab. No woman passes without getting information. ... We know what we are doing. Even our percent right now, we are high. We want it that way. Our documentation is all complete. Any new staff is trained on SAIA. Lab tech, Peri-urban private clinic

A commonly cited facilitator of maintaining SAIA was the low complexity of the intervention. While several clinics identified early barriers, such as difficulties in documenting HTC, by the second year of the study clinic staff expressed that SAIA was easy to conduct.

It is easy. Daily work operations. [We] just did work we're supposed to do ... When you hear about SAIA, you think it's big. It's not. Just knowing your work and doing it for better outcomes. Nurse in-charge, Peri-urban public clinic

Implementation climate was also a facilitator to SAIA continuation. Most clinic staff discussed a willingness among staff to conduct SAIA and satisfaction in seeing positive improvements in HTC as a result.

Staff like it when you see results. The dedication of staff is amazing. ... We know as a facility we want to be champions of … SAIA. Lab tech, Peri-urban private clinic

Staff are dedicated to make SAIA work. Nurse in-charge, Peri-urban public clinic

For some clinics, this sense of commitment extended to leader engagement, with clinic leaders reporting their commitment to SAIA and their involvement to ensure that all staff are properly trained on SAIA components. In contrast, lack of strong engagement from leadership appeared to act as a barrier to sustainability. A clinic that had low HTC performance during Stage 2 reported:

Staff are ready to work if given the tools. We see no problem. … Admin needs to put [in] more effort. We have mentioned about getting [HIV testing] kits but it takes forever to make decisions. Sister in-charge, Urban private clinic

An emerging theme was the systems’ architecture, and the critical role that the Mombasa County DOHS played in successful implementation. Even among high-performing clinics, issues with obtaining HIV-testing kits were prevalent. Testing kits come from the DOHS, and these findings suggest a systems-level issue with provision of commodities. Similarly, staff turnover was cited as a barrier to sustainability. Staff turnover in public facilities is often a result of DOHS-wide reassignments of staff, and this makes it difficult to maintain institutional knowledge of SAIA. However, some aspects of the system were seen as facilitators of continuation, particularly relating to oversight. Sub-county RH and STI coordinators visit the clinics as part of their normal workflow, and embedding SAIA oversight into this structure was seen as a motivator by clinic staff:

Frequent supervision will help, especially from MOH, like they usually do … supervision from [sub-county RH and STI coordinators] really helped to keep us on [our] toes. Nurse in-charge, Per-urban private clinic

Interview responses indicated that at some facilities, SAIA had become part of routine internal operations, suggesting clinics were able to sustain at least some components of SAIA delivery on their own with occasional oversight from DOHS implementers:

We meet every month twice and ensure that all our work including FP clinic and SAIA is meeting objectives. Lab tech, Peri-urban private clinic

Finally, the perceived longer-term sustainability of the SAIA implementation strategy was apparent in many of the responses from clinic staff. Due largely to the facilitators described above, staff expressed the belief that SAIA was effective, easy, and some suggested that it should even be implemented in other settings or with other health outcomes. One staff member stated:

The results are good, and we shall continue doing it even as you say the study is over. Lab tech, Peri-urban private clinic

## Discussion

In Stage 2 of this cluster-randomized controlled trial, 39% of planned DOHS-led SAIA visits occurred, showing moderate ability to sustain supervised SAIA delivery once it was transitioned to County DOHS leadership with minimal support from study staff. This result was influenced by the timing of the study, which was conducted in the context of multiple widespread disruptions in healthcare and short-term clinic closures resulting from the COVID-19 pandemic and a healthcare worker strike. Despite these disruptions, the positive effect of SAIA on HTC rates in family planning clinics enrolled in the intervention arm of the trial was sustained during implementation by the County DOHS. Clinics in the intervention arm consistently sustained high levels of HTC, while control clinics saw declines in both pre-test counseling and testing.

Integration of HTC into family planning clinics has been found to be an effective means of reaching women of childbearing age [3, 4]. A recent systematic review assessing HTC integration into family planning services found an overall increase in HIV testing as a result of integration [3]. In Kenya, a pre-post study in 23 family planning clinics reported success in increasing HTC using a clinic-based implementation strategy targeting provider training [23]. A later study using a non-randomized comparison design to test a similar implementation strategy found an increase in HTC at the clinics implementing the strategy over the course of study follow-up [24]. The results of this two stage trial demonstrate that SAIA could be an effective and sustainable means of increasing HIV testing coverage and knowledge of HIV status among reproductive age women [16].

This study provided valuable insight on the sustainment of the SAIA implementation strategy in the context of family planning clinics in Mombasa County. While DOHS implementers only completed 39% of SAIA oversight visits, interviews with clinic staff suggest that HTC remained high in Stage 2 due to HTC becoming a normalized part of clinic visits, and SAIA becoming a routine activity for some clinics. This suggests successful institutionalization of HTC, likely resulting from the regular implementation of SAIA during Stage 1 of the trial. Institutionalization can occur when the intervention becomes embedded in a system [12, 14, 25]. While there are other pathways to sustainment [26], institutionalization is an effective means to ensure the continuation of programs after the initial research or funding stage has ended. The maintenance of a high level of HTC seen in our study, as well as the results of the family planning staff interviews, suggest effective institutionalization. The patterns observed in pre-test counseling serve as a potential example of this. While there was a significant drop off in counseling in the control clinics during the strike, clinics in the intervention arm maintained their high counseling rate, suggesting that counseling had become a routine part of care even when minimal services were provided.

In addition to the facilitators identified by family planning staff, such as low complexity and a favorable implementation climate, structural aspects of the SAIA implementation strategy and HTC evidence-based intervention were likely important drivers of institutionalization. Previous studies have found that institutionalization is facilitated by permanent funding, repeated reinforcement, and integration into subsystems at the facilities [12, 14, 25]. Both HTC provision and DOHS oversight are funded through Mombasa County. Further, the iterative nature of SAIA allowed for repeated reinforcement. While cost and repetition were not explicitly addressed in staff interviews, they likely played a role in sustainment of the SAIA implementation strategy and the HTC that it targeted in this trial.

Interestingly, our results differed significantly between private and public clinics. At the time of Q8 data collection, the healthcare worker strike was ongoing, impacting public clinics but not private ones. A number of health services offered through public facilities were interrupted due to the strike, including HIV programs, family planning services, cervical cancer screening, and tuberculosis treatment. Within this study, for pre-test counseling, both public and private clinics saw a decline in counseling during Q8 among control clinics, while counseling remained steady at clinics in the intervention arm. This same pattern was observed for HIV testing in private clinics. However, in public clinics, those in the intervention arm experienced a substantial drop in HIV testing during Q8, matching the testing rates of the control clinics. Therefore, when clinics were at a reduced capacity, clinics in which SAIA was an integrated strategy were able to maintain counseling but not HIV testing. This could be driven by clinic-level factors (e.g., reduced capacity to provide testing) or client-level factors (e.g., reduced willingness due to longer wait times). Further research is needed to understand how interruptions in delivery of implementation strategies and the interventions that they target, particularly interruptions resulting from factors outside of the control of the clinic, may impact institutionalization and long-term sustainability.

The timing of this study during the COVID-19 pandemic and healthcare worker strike provided a natural experiment of the sustainment of the SAIA implementation strategy and family planning clinic-based HTC under increased stress on the healthcare system. During the West African Ebola epidemic in 2014, evidence from Sierra Leone and Guinea suggested decreases in HTC, both generally and among women of childbearing age [27, 28]. Moreover, emerging evidence suggests that the COVID-19 pandemic has resulted in interruptions to HIV testing in sub-Saharan Africa [29, 30]. In the present study, a steep drop in number of new family planning clients in Q8 led to a small denominator and reduced power in our sample. However, despite the reduced number of eligible family planning clients, the proportion of HTC uptake remained high in clinics in the intervention arm and starkly contrasted with the drop-off in HTC seen in control clinics, particularly in HIV counseling. These results provide important evidence of the sustainment of the SAIA implementation strategy and its beneficial effect on HTC in the context of multiple major healthcare disruptions. The results suggest that a data-driven approach to systems analysis and improvement may create greater health systems resilience. Future work is needed to directly test this hypothesis and to understand the mechanisms of the effects observed.

In the present study, fidelity was measured as the frequency of SAIA visits led by DOHS staff. One limitation of this analysis is the lack of more granular measures of fidelity, such as adherence to each step of SAIA, adaptations made, and reasons for adaptations. Studies in other contexts provide more detailed insight into SAIA fidelity, as well as the core components of SAIA and how clinics may adapt the strategy [7]. The original SAIA trial was conducted in Mozambique, Kenya, and Cote d’Ivoire to improve PMTCT of HIV. In these contexts, flow mapping and continued quality improvement (CQI) cycles were considered core components of the intervention, while the cascade analysis tool was identified in some settings as being overly complex and nonessential in places with low HIV burden [31]. Similarly, a study that piloted SAIA to improve the pediatric and adolescent HIV care cascade in Kenya found that flow mapping and CQI were compatible with existing workflows, but that the cascade analysis was difficult to use [32]. Building on these findings, several SAIA studies are providing more detailed data on fidelity and adaptation to SAIA designs. The SAIA-SCALE study, testing SAIA for PMTCT services in Mozambique, measured fidelity through a tablet-based survey completed by clinic staff to track number of SAIA cycles, attendants at each cycle meeting, and the number, content, and results of micro-interventions tested [33]. Two additional studies in Mozambique using SAIA for hypertension and mental health services assessed fidelity as number and frequency of SAIA cycles completed [10] and adherence to the 5-step SAIA cycle [34]. All three studies then categorized clinics as high or low performing and used focus group data to assess features of successful implementation. Results of these studies are forthcoming and will provide important evidence about adaptations to SAIA designs in a variety of healthcare settings.

This study had several strengths. The use of a cluster-randomized trial design provides strong causal evidence of the effectiveness of SAIA. Incorporating both private and public institutions allowed for comparison in different types of facilities. The incorporation of a second year of data collection to formally test and measure sustainment of SAIA and HTC was a novel aspect of this study and allowed for rigorous data collection and a combination of both quantitative and qualitative metrics to assess these outcomes. Finally, the timing of this study unintentionally provided the opportunity to test the sustainment of the SAIA implementation strategy and performance of HTC in family planning clinics in the context of widespread healthcare disruptions, which provided a unique perspective on sustainability under extraordinary conditions.

The results should be interpreted in light of a number of limitations. Data on HTC was collected through registries completed by clinic staff, and the delivery of HTC was not independently verified by study staff. Data on the micro-interventions implemented in Stage 2 were not collected, so this could not be analyzed. Our study design did not include an arm that received SAIA in Stage 1 but no DOHS oversight in Stage 2, so we are unable to distinguish how Stage 2 activities impacted sustainment of high HTC in intervention clinics, versus the extent to which this would have occurred regardless of continued SAIA delivery due to institutionalization of HTC established in Stage 1. While we know when SAIA visits were completed, we have limited information about what was done by County DOHS implementers at the SAIA visits, or if clinics conducted SAIA activities (meetings, cascade analysis, flow mapping, and implementation of micro-interventions) in the absence of County implementers. As discussed above, this limits our understanding of SAIA fidelity and what adaptations may have occurred. If SAIA cycles were implemented with low fidelity to the core SAIA components, these unknown adaptations could partially account for the continued high levels of HTC. However, both DOHS implementers and the staff in the family planning clinics were trained in SAIA, and qualitative responses from family planning clinic staff suggest that SAIA was well understood and implemented within clinics. Further, the use of County DOHS implementers could potentially have led to contamination, as these same coordinators were visiting and overseeing control facilities as part of their regular programmatic activities. Finally, due to the healthcare interruptions, fewer new family planning clients attended clinics during the final quarter of analysis, reducing our power to detect associations.

There are a number of future directions for this research. A large scale-up of the SAIA implementation strategy is planned and will provide important evidence of whether these results can be replicated at a programmatic scale. Incorporating data on linkage to care and ART retention could provide valuable insight into the ways in which this implementation strategy impacts the downstream steps of the HIV care cascade and where further efforts to improve implementation are still needed. Expanding the SAIA implementation strategy to also include screening and linkage to pre-exposure prophylaxis (PrEP) could provide added value toward HIV prevention. Further research on sustainability, particularly focused on core components and adaptability, could provide important evidence on what aspects of the SAIA implementation strategy are most critical and how to best integrate them into routine clinical operations.

## Conclusions

These findings show that clinics receiving SAIA sustained improvements in HTC after one year under DOHS leadership, even in the context of wide-scale healthcare disruptions and incomplete maintenance of the implementation strategy. These findings demonstrate promising evidence of the sustainability of systems interventions and provide an example of successful integration of an implementation strategy into DOHS programmatic activities.

## Data Availability

This study was conducted with approval from the Kenyatta National Hospital—University of Nairobi Ethics and Research Committee (KNH-UON ERC), which requires that we release data from Kenyan studies (including de-identified data) only after they have provided their written approval for additional analyses. As such, data for this study will be available from the authors upon request, with written approval for the proposed analysis from the KNH/UON ERC. Their application forms and guidelines can be accessed at http://erc.uonbi.ac.ke/. To request these data, please contact KRTC administrator at kenyares@uw.edu
